# Essential oils of plants and their combinations as an alternative adulticides against *Anopheles gambiae* (Diptera: Culicidae) populations

**DOI:** 10.1038/s41598-022-23554-6

**Published:** 2022-11-09

**Authors:** Mahamoudou Balboné, Ignace Sawadogo, Dieudonné Diloma Soma, Samuel Fogné Drabo, Moussa Namountougou, Koama Bayili, Rahim Romba, Georges Benson Meda, Honorat Charles Roger Nebié, Roch K. Dabire, Imaël H. N. Bassolé, Olivier Gnankine

**Affiliations:** 1 Laboratoire d’Entomologie Fondamentale et Appliquée, Unité de Formation et de Recherche en Sciences de la Vie et de la Terre, Université Joseph KI-ZERBO, 03 BP 7021, Ouagadougou, Burkina Faso; 2 Institut de Recherche en Sciences Appliquées et Technologies, 03 BP 7047, Ouagadougou, Burkina Faso; 3 Institut de Recherche en Sciences de la Santé/Centre Muraz, BP 545, Bobo-Dioulasso, Burkina Faso; 4 Université Nazi Boni, 01 BP 1091, Bobo-Dioulasso, Burkina Faso

**Keywords:** Natural products, Chemical biology, Evolution, Ecology

## Abstract

The persistence of malaria and the increasing of resistance of *Anopheles gambiae* species to chemicals remain major public health concerns in sub-Saharan Africa. Faced to these concerns, the search for alternative vector control strategies as use of essential oils (EOs) need to be implemented. Here, the five EOs from *Cymbopogon citratus, Cymbopogon nardus, Eucalyptus camaldulensis*, *Lippia multiflora*, *Ocimum americanum* obtained by hydro distillation were tested according to World Health Organization procedures on *An. gambiae* “Kisumu” and field strains collected in “Vallée du Kou”. Also, the binary combinations of *C. nardus* (Cn) and *O. americanum* (Oa) were examined. As results, among the EOs tested, *L. multiflora* was the most efficient on both *An. gambiae* strains regarding KDT_50_ (50% of mosquitoes knock down time) and KDT_95_ and rate of morality values. Our current study showed that C8 (Cn 80%: Oa 20%) and C9 (Cn 90%: Oa 10%), were the most toxic to *An. gambiae* strain “Vallée de Kou” (VK) with the mortality rates reaching 80.7 and 100% at 1% concentration, respectively. These two binary combinations shown a synergistic effect on the susceptible population. However, only C9 gave a synergistic effect on VK population. The bioactivity of the two EOs, *C. nardus* and *O. americanum*, was improved by the combinations at certain proportions. The resistance ratios of all EOs and of the combinations were low (< 5). The combinations of *C. nardus* and *O. americanum* EOs at 90: 10 ratio and to a lesser extent *L. multiflora* EO, could be used as alternative bio-insecticides against malaria vectors resistant to pyrethroids in vector control programmes.

## Introduction

Malaria remains one of the world’s deadliest diseases. It is a life-threatening disease caused by parasites that are transmitted to people through the bites of infected female Anopheles mosquitoes. Each year, 154 to 289 million persons are infected with 490–836 thousand deaths recorded mostly in children under 5. About 90% of this burden is recorded in Africa^1^. In Burkina Faso, malaria is a major health issue and accounts for 43% of medical consultations and 22% of deaths were recorded. This country is among the 10 most affected (3% of cases and 4% of deaths worldwide)^2^ by malaria disease.

Until now, long-lasting insecticidal nets (LLINs) and indoor residual spraying (IRS) remain the two main tools commonly used in controlling adults of *Anopheles gambiae*, the vector of malaria^3^. Pyrethroids are only products used for LLINs^4^. However, pyrethroid resistance is a threat to the efficiency of these protective tools, especially when resistance is occurred at high levels^5,6^. In Burkina Faso, pyrethroid resistance has been observed throughout the country for several years and still a concern because the use of pyrethroid-based LLINs does not provide the expected levels of individual and community protection^7^.

To improve the effectiveness of vector control tools, the World Health Organization (WHO) has developed a global plan for the management of insecticide resistance (GPIRM)^8^. Key elements of this plan include: (1) insecticide rotation; (2) mixtures of at least two different insecticides; (3) alternate use of at least two insecticides of different classes; and, (4) mosaic use of insecticides. Today, a limiting factor in the development of these strategies were the absence of alternative classes of insecticides for LLINs. However, in recent years, several studies have shown that the use of piperonyl butoxide (PBO) as a synergist restores susceptibility to deltamethrin or permethrin in several regions of the country^9,10^.

To manage the resistance of insects generally and mosquitoes specifically to chemical insecticides, many research programmes have focused on natural products derived from plants as an alternative to conventional insecticides used in vector control for which resistance has been detected^11^. Among the many natural products, essential oils (EOs) and their constituents have received considerable attention in the search for new pesticides and have been found to possess insecticidal properties^12^.

EOs from plants are secondary metabolites comprising different bioactive compounds and have gained importance in terms of alternative to chemicals. They are biodegradable, environmentally safe and easy to use and compose of a mixture of different bioactive compounds which offer less chance for emerging resistance^13^.

Previous studies have identified more than 3000 compounds from 17,500 aromatic plants^14^. Most of them have been tested for their insecticidal properties and have been reported to have insecticidal effects^15,16^. Other studies have shown equal or higher toxicity of the major compounds compared to its whole EO^17^. Some studies have also attempted to formulate efficient insecticides by combining different plants with chemical insecticides to increase the overall toxicity and minimize the secondary effects^18^. Other studies have shown insecticidal properties of EOs of *Cymbopogon nardus*, *Eucalyptus camaldulensis*, *Lippia multiflora*, and *Ocimum americanum*^19^. These studies focused on *Aedes aegypti* populations in the city of Ouagadougou, Central Burkina Faso. Although recent studies have shown that combinations of EO of *L. multiflora* and *Cymbopogon schoenanthus* gave synergistic effects against mosquitoes from the *An. gambiae* populations Kisumu strain^20^**,** little is known about the effect of EOs mixture on resistant *An. gambiae* populations.

The objective of the current study aimed at examining the adulticidal activities of EOs of five aromatic plants of *Cymbopogon citratus, Cymbopogon nardus*, *Eucalyptus camaldulensis, Lippia multiflora and Ocimum americanum*, on susceptible and resistant *An. gambiae* populations collected in “Vallée du Kou” (Bama), Western Burkina Faso. ﻿They were chosen firstly due to endogenous data provided from informants questioned in areas investigated as well as the data from the literature. This current study, also evaluate the toxicity level of binary combinations of EOs of *C. nardus* and *O. americanum*, on the two *An. gambiae* populations.

## Materials and methods

### Sampling of larvae and rearing

Larvae and pupae of the resistant strain of *Anopheles gambiae* “VK” were collected from June to October 2021, in the “Vallée du Kou” (Bama). The frequency of the *kdr L1014 F* mutation in this strain was closed to fixation (F = 0.99). The "Vallée du Kou" (VK) is a district of the Bama department (11º 23′ 59'' N, 4º 25′ 46'' W) in the province of Houet, the economic capital of Burkina Faso. The larvae were brought and reared in the insectarium of the “Institut de Recherches en Sciences de la Santé/Direction Régionale de l’Ouest” (IRSS/DRO) located in Bobo-Dioulasso. The larvae were fed with tetraMin (Tetrawerke, Melle, Germany). Adult mosquitoes emerging from the pupae of the collected strain were placed in cages and fed with 10% sugar solution. Female mosquitoes of the resistant strain were used for susceptibility tests. It is the same for the susceptible strain of *An. gambiae* "Kisumu" maintained at the insectary, and used as a reference strain in this current study.

Mosquitoes were reared at a temperature of 27 ± 2 °C, a relative humidity of 70 ± 5%, 12 h of light and 12 h of darkness.

### Essential oils extraction

The essential oils were obtained from the leaves of five aromatic plant species, *Cymbopogon citratus* (DC.) Stapf, *Cymbopogon nardus* (Linn.) Rendle, *Eucalyptus camaldulensis* Dehnh., *Lippia multiflora* Moldenke, and *Ocimum americanum* (Willd.) A.J. Paton*.* The collection of these plants was done at botanical garden located in “Institut de Recherche en Sciences Appliquées et Technologies” (IRSAT). They were extracted at the IRSAT by hydrodistillation (HD) using a *clevenger*-type apparatus and stored in a dark glass bottle at 4 °C prior to use. The EO extraction process has been described following the protocol described by Drabo et *al*.^21^. The combinations of two EOs were made, after having done the bioassay tests with the whole EOs. Table 1 shows all combinations and concentrations tested.

**Table 1:** Combination codes with proportions of the two essential oils.

Codes essential oils	C1	C2	C3	C4	C5	C6	C7	C8	C9
Cn (%):	10	20	30	40	50	60	70	80	90
Oa (%):	90	80	70	60	50	40	30	20	10

Cn, *C. nardus*; Oa, *O. americanum;* %, percentage.

### Analysis by gas chromatography/flame ionization detector (GC/FID)

Gas chromatography/flame ionization detector (GC-FID) analysis of essential oils of *C. citratus, C. nardus, E. camaldulensis, L. multiflor*a and *O. americanum* obtained from their leaves was performed on an Agilent 6890 N GC instrument equipped with a FID, with a narrow bore DB-5 column (length 10 m, inner diameter 0.1 mm, film thickness 0.17 mm; Agilent, Palo Alto, CA) according to protocol previously used by Drabo et *al.*^21^. The oven temperature was programmed from 60 ℃ to 165 ℃ at 8 ℃/min and from 165 ℃ to 280 ℃ at 20 ℃/min, with 1 min of post-operation at 280 ℃. Diluted samples (1/100 in sample) were subjected to an ionization test. Diluted samples (1/100 in acetone) of 1.0 µl were injected manually and without fractionation. The percentage peak area was calculated on the basis of the FID signal using the GC HP-Chemstation software (Agilent Technologies).

### Gas chromatography/mass spectrometry (GC/MS) analysis

GC/MS analysis was performed on a GC HP 6890 coupled to MSD HP 5972 (Hewlett Packard, Palo Alto, CA), and was equipped with a ZB-5MS Zebron capillary column (length 30 m, ID 0.25 mm, film thickness 0.25 mm; Agilent). The carrier gas used was helium and the oven temperature were maintained at 45 °C for 2 min and then increased from 45 ℃ to 165 °C (4 °C/min) and then from 165 ℃ to 280 °C (15 °C/min).

### Bioassays on adult mosquitoes

Susceptibility tests were carried out using WHO insecticide susceptibility test-kits and standard procedures^3^. Impregnation of Whatman n°1 papers were done according to the protocol adopted by N'Guessan et al^22^ and WHO^23^ standard procedure. Four rectangular papers (size 12 cm × 15 cm) were impregnated with 2 ml of a given concentration of an essential oil/combination of EOs diluted in acetone at varying proportions. Three concentrations (0.1%, 0.5% and 1%), prepared using serial dilutions (v/v) of EOs in acetone, were used for each EO or combination of *Cymbopogon nardus* (Cn) and *Ocimum americanum* (Oa).

For combination of EOs and for each concentration (0.1; 0.5 and 1%), 9 binary combinations were done (Table 1).

Control papers were impregnated with 2 ml of acetone only. Tests were carried out at 25 °C (± 2 °C) and 70–80% relative humidity (RH). The number of mosquitoes knocked down was recorded every 5 min. Permethrin 0.75% was used as positive control insecticide. After the exposure time, mosquitoes were transferred to holding tubes and were fed with 10% sugar juice for 24 h. Subsequently, the mortality was recorded. The susceptible strain *Anopheles gambiae* “Kisumu” was used as reference to determine the diagnostic concentrations.

### Data analysis

The data obtained from the bioassays performed were analyzed using XLSTAT statistical software version 2015.1.01. The knock-down time (KDT_50_ and KDT_95_), lethal concentrations (LC_50_ and LC_99_) and 95% confidence limits (95% CL) were calculated by probity analysis using the same statistical software in order to compare the toxicity of the plant EOs against the tested mosquito adults. The KDT_50_, KDT_95_, CL_50_, CL_99_ values and mortalities were considered significantly different between the EOs (p < 0.05) if the 95% CL (Confident Limits) did not overlap. In all tests, no control mortality was detected after the 24-h exposure; therefore, no correction was required based on Abbot's formula^24^.

According to WHO^23^ criteria, mosquitoes populations were “resistant” if less than 90% mortality was observed, “suspected resistant” if mortality rates were between 90 and 98% and “susceptible” for more than 98% mortality rate.

Interactions between the combinations performed were determined using the Fractional Inhibitory Concentration indices or FIC indices. These indices were calculated in the following way: FIC indice = FIC_A_ + FIC_B_ and FIC_A_ = MIC_AB_/MIC_A_ and FIC_B_ = MIC_AB_/MIC_B_ where FIC_A_ and FIC_B_ are the minimum inhibitory concentrations that kill 50% of adult mosquitoes for EO A and B respectively.

Thus, we have (1) FIC_A_: Fractional Concentration of A; (2) FIC_B_: Fractional Concentration of B; (3) MIC_AB_: Minimum Inhibitory Concentration of A or B in the combination; (4) MICA: Minimum Inhibitory Concentration of A; (5) MIC_B_: Minimum Inhibitory Concentration of B.

The results were interpreted as follows: (1) Synergy: FIC < 0.5; (2) additive: 0.5 ≤ FIC ≤ 1; (3) indifferent: 1 ≤ FIC ≤ 4; or (4) antagonism: FIC > 4^25,26^.

The diagnostic concentration was obtained from twice the LC_99_ on susceptible strain^3^. The resistance ratio (RR) between the VK and Kisumu strains was calculated by dividing the LC_50_ of the VK strain by the LC_50_ of the Kisumu strain. According to WHO^27^, RR < 5 indicates low resistance, RR 5–10 denotes moderate resistance, and RR > 10 indicates high resistance.

### Statement on research involving plant species

The collection of targeted plants was done at botanical garden located in “the Institut de Recherche en Sciences Appliquees et Technologies (IRSAT)”, in Ouagadougou, Burkina Faso and comply with institutional, national and international guidelines and legislation.

Before extracting essential oils, *Cymbopogon citratus, Cymbopogon nardus, Lippia multiflora, Eucalyptus camaldulensis*, *Ocimum americanum* plants were identified by Cyrille SINARE and specimen were deposited in herbarium of “laboratoire de Biologie et Ecologie vegetale” of Joseph KI-ZERBO University, Ouagadougou, Burkina Faso as under ID number: :17949; 17950; 17951; 17971; 17988 respectively.

## Results

### Chemical composition of the essential oils

The main compounds of the 5 essential oils are summarized in Table 2. The EO of *C. citratus*, consisting only of oxygenated monoterpenes (99.9%) which were neral (44.7%) and geranial (55.2%). The EO of *C. nardus* consisted of six compounds mostly oxygenated monoterpenes (77.9%), characterized by citronellal (41.7%), geraniol (20.8%) and β-elemene (11%). As for EO of *E. camaldulensis,* it consisted of sixteen compounds mainly hydrocarbon monoterpenes, rich in 1,8-cineole (59.5%). The EO of *L. multiflora* consisted of sixteen compounds dominated by monoterpenes (69.51%), characterized by β-Caryophyllene (20.1%), p-cymene (14.6%), thymol acetate (12.0%) and 1.8 cineol (11.6%) whereas that of *O. americanum* consisted of twenty-six (26) compounds characterized by a high percentage of 1.8-cineole (31.22%) followed by camphor (12.73%).

**Table 2:** Major compounds of the 5 essential oils tested on adults of *Anopheles gambiae.*

Essential oils	Major compounds	Retention indices	Mono Hydro (%)	Mono Oxy (%)	Sesqui Hydro (%)	Sesqui Oxy (%)
*C. citratus*	Geranial	1268	–	55.2	–	–
	Neral	1242	–	44.7	–	–
	Total other compounds		–	–	–	–
*C. nardus*	Citronellal	1158	–	41.7	–	–
	Geraniol	1253	–	20.8	–	–
	β-Elemene	1372	–	–	11	–
	Total other compounds		–	15.4	3.7	–
*E. camaldulensis*	1,8 cineol	1034	–	59.55	–	–
	Total other compounds		24.89	7.41	1.4	0.34
*L. multiflora*	β-caryophyllene	1415	–	–	20.1	–
	p-cymene	1027	14.6	–	–	–
	Thymul acetate	1355	–	12	–	–
	1.8 cineol	1034	–	11.6	–	–
	Total other compounds		18.41	12.9	5.7	4.5
*O. americanum*	1,8 cineol	1034	–	31.22	–	–
	Camphor	1151	–	12.73	–	–
	Total other compounds		29.59	43.95	13.03	0.99

Mono : monoterpenes ; Sesqui : sesquiterpenes, Hydro : Hydrocarbon and oxy : Oxygenated.

### Knock-down times (KDT) and adulticidal effects of EOs and combinations

*Lippia multiflora* EO alone exhibited high toxicity with LC_50_ values of 0.21 and 0.67%, LC_99_ values of 0.74 and 1.17%, respectively (Table 3) and also with a rate of mortality of 100 and 96.88% at concentration (1%) on the susceptible Kisumu strain and the field strain VK, respectively (Table 4). This EO exhibited the lowest KDT_50_ and KDT_95_ values on both *An. gambiae* strains tested (19.8 and 87.2 min on Kisumu, 58.8 and 142.7 min on VK, respectively) (Table 4).

**Table 3:** LC_50_, LC_99,_ diagnostic concentration and resistance ratios for all essential oils and combinations of *C. nardus* and *O. americanum* tested on *Anopheles gambiae* populations.

EOs/ Combinations	*Anopheles gambiae "Kisumu"*		*Anopheles gambiae "VK"*		Diagnostic concentration (%)	Resistance ratios (RR)	
	LC_50_ (%) (CL95)	LC_99_ (%) (CL95)	LC_50_ (%) (CL95)	LC_99_ (%) (CL95)		RR_50_	RR_99_
*C. citratus*	2.3	4.7	2.7	4.84			
	(1.9–2.7)	(4.4–6)	–	–	9.4	1.17	0.97
*C. nardus*	0.63	1.66	1.12	2.16			
	(0.6–0.7)	(1.4–2)	(1–1.3)	(1.8–2.7)	3.32	1.78	0.77
*E. camaldulensis*	2.86	5.26	2.94	5.05			
	(1.6–9.3)	(4.1–5.5)	–	–	10.1	1.028	1.04
*L. multiflora*	0.21	0.74	0.67	1.17			
	0.16–0.3	(0.6–0.9)	(0.6–0.7)	(1.1–1.3)	1.48	3.19	0.63
*O. americanum*	2.18	4.13	2.21	4.59			
	(1.5–3)	(2.6–5.5)	(1.5–3.5)	(2.9–5.8)	9.18	1.01	0.9
C1 (Cn 10%: Oa 90%)	1.37	2.95	2.9	6.4			
	(1.1–1.8)	(2.3–3.4)	–	–	5.9	2.12	0.46
C2 (Cn 20%: Oa 80%)	0.76	1.69	1.18	2.45			
	(0.7–0.8)	(1.5–2)	(1.0–1.4)	(2–3.3)	3.38	1.55	0.69
C3 (Cn 30%: Oa 70%)	0.61	1.31	2.07	5			
	(0.5–0.7)	(1.2–1.5)	(1.5–5)	(4.2–5.9)	2.62	3.39	0.26
C4 (Cn 40%: Oa 60%)	0.46	1.19	1.25	2.48			
	(0.4–0.5)	(1–1.4)	(1.2–1.5)	(2–3.4)	2.38	2.72	0.48
C5 (Cn 50%: Oa 50%)	0.57	1.16	1.36	3.26			
	(0.5–0.6)	(1–1.3)	(1.1–2)	(2.4–5.5)	2.32	2.38	0.35
C6 (Cn 60%: Oa 40%)	0.28	1	1.08	2.15			
	(0.2–0.3)	(0.9–1.2)	–	–	2	3.86	0.46
C7 (Cn 70%: Oa 30%)	1.93	4.3	2.44	5.07			
	(1.4–4)	(3–4.7)	(1.6–3.2)	(3.1–5.6)	8.6	1.26	0.85
C8 (Cn 80%: Oa 20%)	0.23	0.58	0.89	1.3			
	(0.2–0.3)	(0.5–0.7)	(0.8–1)	(1.28–3)	1.16	3.87	0.45
C9 (Cn 90%: Oa 10%)	0.22	0.52	0.32	0.59			
	(0.2–0.3)	(0.4–0.6)	(0.3–0.4)	(0.5–0.7)	1.04	1.17	0.97

LC_50_: lethal concentration for 50% mortality; CL_95=_ Confidence limit at 95%; Cn : *Cymbopogon nardus*; Oa : *Ocimum americanum;* % : percentage; RR_50_ : Resistance ratios with LC_50_; VK : Vallée du Kou.

**Table 4:** KDT_50_, KDT_95_ and rates of mortality of essential oils tested on the susceptibility (Kisumu) and field (VK) strains of *Anopheles gambiae.*

Essential oils	Concentrations (%)	Anopheles gambiae « VK »			Anopheles gambiae « VK »		
		KDT_50_ (min)	KDT_95_ (min)	Mortality (%)	KDT_50_ (min)	KDT_95_ (min)	Mortality (%)
*C. citratus*	0.1	103.24	204.66	11.05	140.2	318.7	2.15
	0.5	77.10	155.86	17.35	124.9	275.8	3.33
	1	60.19	132.39	35.29	116.5	233.6	4.76
*C. nardus*	0.1	88.37	196.99	18.48	148.6	241.8	2.15
	0.5	33.3	108.9	26.09	90.2	149.5	15.21
	1	5.8	11.5	86.36	12.4	70.7	40.63
*E. camaldulensis*	0.1	192.2	289.7	1.05	227.3	309.7	0
	0.5	157.1	275.1	6.45	189.9	286.7	1.12
	1	144.8	266.5	9.78	166.1	280	3.16
*L. multiflora*	0.1	19.8	58.8	30.30	87.2	142.7	13.16
	0.5	10.5	19	90	32	57.7	63.56
	1	−28.3	−8.2	100	−5.9	1.5	96.88
*O. americanum*	0.1	137.5	225.6	1.10	156.4	267.5	0
	0.5	87.06	146.64	6.73	144.7	248.9	2.2
	1	30.04	60.76	41.49	116.3	204.6	6.38
Permethrin	0.75	16.3	30	100	41.2	90.4	62.5

KDT_50_: 50% of mosquitoes knock down time; KDT_95_: 95% of mosquitoes knock down time; VK : Vallée du Kou;

%: percentage; min : minutes.

*E. camaldulensis* EO was the least toxic with rate of mortality above 10% at  concentration (1%) on the susceptible Kisumu strain (Table 4), KDT_50_ values of 144.8 min on the Kisumu strain, 166.1 min on the VK strain and LC_50_ of 2.86 and 2.94%, LC_99_ of 5.05 and 5.26% on Kisumu and VK strains, respectively. Moreover, EOs of *C. nardus* (LC_50_ of 0.63 and 1.12% on Kisumu and VK, respectively) and *O. americanum* (LC_50_ of 2.18 and 2.21% on Kisumu and VK, respectively) were more toxic than *C. citratus* (LC_50_ of 2.3 and 2.7% on Kisumu and VK, respectively) (Table 3). For the susceptible strain Kisumu, no difference was observed between EOs of *C. citratus*, *E. camaldulensis* and *O. americanum* regarding the LC_50_ and LC_99_. However, for the field strain VK, the confidence limits at 95% for the LC_50_ and LC_99_ do not overlap for all OEs tested (Table 3).

Regarding the combinations on both *An. gambiae* strains (Kisumu and VK), the C9 combination exhibited high toxicity on all strains. Indeed, this combination shown a rate of mortality reaching 100% at concentration (1%) on both strains (Table 5). Conversely, with Permethrin, the rate of mortality reached 62.5% confirming the presence of phenotypic resistance on VK populations. In addition, C9 was the only combination that produced a synergistic effect on both strains (Tables 6 and 7). Also, this combination exhibited the lowest KDT values (Table 5) and the lowest LC_50_ and LC_99_ values on both strains (0.22 and 0.52% with Kisumu, 0.32 and 0.59% with VK) (Tables 5).

**Table 5:** KDT50, KDT95 and rates of mortality of essential oils combinations of *C. nardus* (C.n) and *O. americanum* (O.a) tested on the susceptibility (Kisumu) and field (VK) strains of *Anopheles gambiae* populations.

Codes	Combination of essential oil	Concentrations (%)	*Anopheles gambiae "Kisumu"*			*Anopheles gambiae "VK"*		
			KDT_50_ (min)	KDT_95_ (min)	Mortality (%)	KDT_50_ (min)	KDT_95_ (min)	Mortality (%)
C1	Cn 10% : Oa 90%	0.1	102	132.9	3.61	171.6	321.3	2.53
		0.5	9.6	22.1	18.49	33	57.3	16.17
		1	4.4	13.2	29.79	27.4	50.3	19.78
C2	Cn 20% : Oa 80%	0.1	131.7	232.7	1.94	222	310	1.15
		0.5	3.3	13.2	32.29	35	73.3	13.25
		1	2.4	10.2	68.97	16	25.9	26.08
C3	Cn 30% : Oa 70%	0.1	142.8	232.7	6.66	178.1	304	6.25
		0.5	10.8	21	32.04	25	38.5	13.68
		1	5.5	13.4	91.67	16	16.1	20.21
C4	Cn 40% : Oa 60%	0.1	108.7	147.1	22.06	142.4	172.1	4.1
		0.5	7.3	16.9	78.49	20.3	47.6	17.11
		1	6.7	12.4	98.78	7.7	14.3	42.39
C5	Cn 50% : Oa 50%	0.1	91	153.6	4.55	106.7	182.8	3.66
		0.5	11.2	18.6	28.89	23.5	78.7	19.48
		1	0.5	13.1	97.78	7.1	16.5	30.38
C6	Cn 60% : Oa 40%	0.1	77.4	145	19.39	116.1	168.1	7.08
		0.5	15.7	37.6	91.67	48.1	64.9	22.38
		1	8.6	13.4	98.9	8.9	20.7	45.21
C7	Cn 70% : Oa 30%	0.1	131.9	245.3	3.49	166.1	303.3	1.19
		0.5	48.7	99.6	17.84	137.2	225	15.21
		1	11.6	17.4	37.98	13.2	28.8	19.62
C8	Cn 80% : Oa 20%	0.1	76.4	150.6	15.12	119	218.2	8.75
		0.5	−25.2	28.8	95.65	24.2	52.7	27.38
		1	−26.9	1.4	100	−2.1	12.2	80.7
C9	Cn 90% : Oa 10%	0.1	23.5	77	13.98	53.5	126.7	12.15
		0.5	−32.3	3.7	96.7	−5	7.4	94
		1	−227.9	−70.1	100	−227.3	−69.6	100
Permethrin		0.75	16.3	30	100	41.2	90.4	62.5

KDT_50_: 50% of mosquitoes knock down time; KDT_95_: 95% of mosquitoes knock down time; VK: Vallée du Kou; % : percentage; min : minutes; Cn : *Cymbopogon nardus*; Oa : *Ocimum americanum.*

**Table 6:** Effects of combinations of essential oils of *Cymbopogon nardus* (Cn) and *Ocimum americanum* (Oa) on adults of *Anopheles gambiae* susceptible strain (Kisumu) and type of interaction (n = 125 adult).

Combinations of essential oils (%)	Codes	LC_50_ (%)	FIC_Cn_	FIC_Oa_	FIC	Effect
Cn 0% : Oa 100%	Oa	2.18	–	–	–	–
Cn 10% : Oa 90%	C1	1.37	2.17	0.63	2.80	No effect
Cn 20% : Oa 80%	C2	0.76	1.21	0.35	1.55	No effect
Cn 30% : Oa 70%	C3	0.61	0.97	0.28	1.25	No effect
Cn 40% : Oa 60%	C4	0.46	0.73	0.21	0.94	Additive
Cn 50% : Oa 50%	C5	0.57	0.90	0.26	1.17	No effect
Cn 60% : Oa 40%	C6	0.28	0.44	0.13	0.57	Additive
Cn 70% : Oa 30%	C7	1.93	3.06	0.89	3.95	No effect
Cn 80% : Oa 20%	C8	0.23	0.37	0.11	0.47	Synergistic
Cn 90% : Oa 10%	C9	0.22	0.35	0.10	0.45	Synergistic
Cn 100% : Oa 0%	Cn	0.63	–	–	–	–

LC_50_: lethal concentration for 50% mortality FIC: FIC_Cn_ + FIC_Oa_; Synergistic: FIC < 0.5; Additive; 05 ≤ FIC ≤ 1; No effect: 1 ≤ FIC ≤ 4; Antagonistic: FIC > 4; Cn: *Cymbopogon nardus*; Oa : *Ocimum americanum;*

% : percentage.

**Table 7:** Effects of combinations of essential oils of *Cymbopogon nardus* (Cn) and *Ocimum americanum* (Oa) on adults of *Anopheles gambiae* field strain of VK and type of interaction (n = 125 adult).

Combinations of essential oils (%)	Codes	LC_50_ (%)	FIC_Cn_	FIC_Oa_	FIC	Effect
Cn 0% : Oa 100%	Oa	2.21	–	–	–	–
Cn 10% : Oa 90%	C1	2.9	1.31	2.59	3.9	No effect
Cn 20% : Oa 80%	C2	1.18	0.53	1.05	1.58	No effect
Cn 30% : Oa 70%	C3	2.07	0.94	1.85	2.79	No effect
Cn 40% : Oa 60%	C4	1.25	0.56	1.12	1.68	No effect
Cn 50% : Oa 50%	C5	1.36	0.61	1.2	1.81	No effect
Cn 60% : Oa 40%	C6	1.08	0.49	0.96	1.45	No effect
Cn 70% : Oa 30%	C7	2.44	1.1	2.17	3.27	No effect
Cn 80% : Oa 20%	C8	0.89	0.40	0.79	1.19	No effect
Cn 90% : Oa 10%	C9	0.32	0.14	0.28	0.42	Synergistic
Cn 100% : Oa 0%	Cn	1.12	–	–	–	–

LC_50_: lethal concentration for 50% mortality FI : FIC_Cn_ + FIC_Oa_; Synergistic: FIC < 0.5; Additive; 05 ≤ FIC ≤ 1; No effect: 1 ≤ FIC ≤ 4; Antagonistic: FIC > 4; Cn : *Cymbopogon nardus*; Oa : *Ocimum americanum;*

% : percentage.

Moreover, the combinations C6 and C8 exhibited high toxicity with rates of mortality above 98% at concentration (1%) on the Kisumu strain while on VK strain, the toxicity of these two combinations was 45.21 and 80.7% at concentration (1%). With these two combinations, the KDT values were slightly low on both strains (on strain VK: KDT_95_ = 20.7 min 12.2 min at the 1% concentration for C6 and C8, respectively) and, the LC_50_ and LC_99_ were low on both strains (LC_50_ = 1.08 and 0.89%, LC_99_ = 1.3 and 2.15%, respectively).

On the Kisumu strain, C8 produced a synergistic effect while C6 shown an additive effect (Table 6). The lowest diagnostic concentrations of 1.04%, 1.16% and 1.48% were obtained with C9 and C8 and *L. multiflora* EO, respectively (Table 3).

The LC_50_ obtained with the C8 combinations on VK strain showed no difference with those obtained with *C. nardus* EO with no overlapping confidence intervals. As well, the LC_50_ obtained with C3, C4, C5 and C7 showed no difference with those obtained with *O. americanum* EO on VK strain (Table 3). The resistance ratios obtained with all EOs as well as those of all combinations were below 5 (Table 3).

## Discussion

Up to now, the management of insecticide resistance remains a major challenge to achieving effective malaria elimination^1^. Indeed, pyrethroid resistance has been reported in 27 sub-Saharan African countries, raising the urgency of finding alternatives to these insecticides^28,29^. Searching for alternatives to chemical insecticides based on plant extracts may constitute new avenues for controlling malaria mosquito vectors populations.

Here, we evaluate the toxicity level of *C. citratus, C. nardus, E. camaldulensis, L. multiflora* and *O. americanum* EOs. Also, the binary combinations of *C. nardus* and *O. americanum* EOs were examined in terms of toxicity.

Overall, almost all the EOs tested have shown an adulticidal effect on the susceptible (Kisumu) and on the resistant (VK) strain of *An. gambiae*. This insecticidal effect, which is highlighted through knock-down effects, lethal concentrations (LC) and rates of mortality (%) of the adult mosquitoes tested, varied significantly according to the concentrations and kind of EOs used. Our study confirmed the presence of resistance to permethrin in *Anopheles* populations that raise the question of its use in vector control strategies.

Among the EO tested, that of *L. multiflora* remains the most toxic on the two strains of *An. gambiae* tested followed by *C. nardus.* The least toxic EO was *E. camaldulensis*. Interestingly, *L. multiflora* may constitute an alternative regarding the rate of mortality reaching 96.88% in comparison with that found with permethrin. The toxicity of EO of *L. multiflora* in the current study was significantly better than that of *Lantana camara, Hyptis spicigera, Hyptis suaveolens* EOs evaluated on *An. gambiae* strains from Kisumu and fields by Wangrawa et *al.*^30^.

The difference observed in toxicity between the EOs tested in our investigations could be explained by their chemical composition. Indeed, several previous studies had shown that the bioactivity of an EO is attributed to the major compounds as they may constitute the most important part of the total compounds of the EO^15,31,32^. Hence, the toxicity of *L. multiflora* could be explained by its major compounds which are ß caryophyllene, p-cymen, thymol acetate and 1.8 cineol. According to previous studies done by Bassolé et al.^33^, the toxicity of *L. multiflora* EO on *Anopheles gambiae* and *Aedes aegypti* larvae was due to the presence of three major components: thymol, p-cymene and thymol acetate. *Hyptis suaveolens* EO containing also β-caryophyllene and 1.8 cineol^34^ has a high insecticidal activity^35^. Thus, the high insecticidal activity of *L. multiflora* EO was attributed to β-caryophyllene and 1.8 cineol which are major compounds, in the current study. The low insecticidal activity of *E. camaldulensis* EO could be explained probably by the absence of β-caryophyllene. Here, *C. nardus* EO do show intermediate adulticidal effect on *An. gambiae* populations tested unlike that found by Zulfikar^36^. According to these authors, the highest bioactivity from *C. nardus* was due to the presence of geraniol, the same compound found also in *C. nardus* tested in our current study. In fact, no effect was detected with *C. nardus.* The EOs which were less efficient as adulticides in the current study could be efficient as repellents. Indeed, previous studies shown the repellent effect of these EO on mosquitoes^37–39^. The KDT values of these EO mainly at the concentration of 1% could explain this repellent property highlighted by irritant activity.

In addition to their adulticide activity, *L. multiflora* exhibits also toxic effect on larval populations of *An. gambiae* from the same locality^40^.

Overall*, C. nardus* and *O. americanum* EOs provide intermediate rate of mortality*.* Do their combinations may improve the bioefficiency against *An. gambiae* populations? For this purpose, combinations from C1 to C9 were made, each concentration combining a certain proportion of each EO.

Globally, all combinations of the 2 EOs have improved the overall efficiency of the EOs compared to individual EOs. In the current study, the improvement of the adulticidal potential by the combination of the two EOs depends on their ratio in the combination. Indeed, combinations containing 60%, 80% and 90% of *C. nardus* EO were more effective than *C. nardus* EO tested alone. This is in agreement with the work of Bekele and Hassanali^41^; Pavela^42^ who had reported that the biological activity of EOs depends not only on their qualitative composition, but also on the quantitative ratio of their constituents.

Improved efficiency was observed when knock down times were reduced and also synergistic and additive effects were observed for the combinations where the proportions of *C. nardus* EO reach 90%, 80% and 60% and 40%, respectively. This improved toxicity by the combinations of the 2 EOs, could be explained by the combined toxic effect of the major compounds. Previous works have showed that the toxic action of EOs is due to the combined effects of different components, with or without significant individual toxic action against insects^43,44^. According to Burt^45^, individual EOs contain complex components that, when combined with each other, can lead to indifferent, additive, synergistic or antagonistic effects. In an earlier study, Abbassy et al^46^ reported that, in some cases, the whole EO may have a higher insecticidal activity than its isolated major components. For these authors, the minor compounds were essential for the bioefficiency and could allow a synergistic effect or potential influence.

The combinations showing a synergistic or additive effect would have, both some major compounds of the EOs of *C. nardus* and *O. americanum* coupled with a variety of minor compounds. Previous studies have shown that the bioactivity of the EO is a consequence of interaction between the major components, but also other compounds, eventually oligoelements explaining combined effects, additive action between chemical classes and synergism or antagonism^33,47,48^. Generally, it seems that the effect of an active compound can be boosted by other major compounds and/or stimulated by minor compounds to give additive or synergistic effects^49^. Therefore, according to Berenbaum and Neal^50^, minor components present in low percentages can act as synergists, enhancing the bio-efficiency of major constituents by various mechanisms.

Interestingly, among different combinations tested, only the C9 combination provided a synergistic effect on the resistant *An. gambiae* population. Earlier studies had shown that some compounds in EOs could interact to create a synergistic or antagonistic effect according to the ratios of the different EOs in the combination^51,52^. In addition, the appearance of new compounds in the combination of some EOs that do not exist in individual EOs^20,53^ could explain the synergistic effect of C9 combinations. Further studies will evaluate this combination on a large wild *Anopheles* population to assess the phenotypic data as well as determination of their components. Also, other combinations including *L. multiflora* need to be evaluated on mosquitoes’ populations.

## Conclusion

The current study confirmed that the essential oils of *Cymbopogon citratus, Cymbopogon nardus, Eucalyptus camaldulensis, Lippia multiflora* and *Ocimum americanum* have insecticidal properties. *L. multiflora* EO was efficient in comparison with the others tested. Our current study showed that the activity of the two combined EOs, *C. nardus* and *O. americanum*, was improved by the combinations at certain proportions regarding the values of rate of mortality reaching at least 98%.

The EO of *L. multiflora* and combinations of EO of *C. nardus* and *O. americanum* could be valuable alternatives in the malaria vector control.

## Data Availability

The datasets generated and analyzed during the current study are available from the corresponding author on reasonable request. Requests to access these datasets should be directed to the corresponding author.
